# Quercetin Abrogates Oxidative Neurotoxicity Induced by Silver Nanoparticles in Wistar Rats

**DOI:** 10.3390/life12040578

**Published:** 2022-04-13

**Authors:** Samar S. Elblehi, Eman M. Abd El-Maksoud, Adil Aldhahrani, Saqer S. Alotaibi, Heba I. Ghamry, Salwa A. Elgendy, Mohamed Mohamed Soliman, Mustafa Shukry

**Affiliations:** 1 Department of Pathology, Faculty of Veterinary Medicine, Alexandria University, Alexandria 22758, Egypt; samar.saad@alexu.edu.eg; 2 Department of Biochemistry, Faculty of Veterinary Medicine, Alexandria University, Alexandria 22758, Egypt; eman.abdelmaksoud@alexu.edu.eg; 3 Clinical Laboratory Sciences Department, Turabah University College, Taif University, Taif 21995, Saudi Arabia; a.ahdhahrani@tu.edu.sa (A.A.); mmsoliman@tu.edu.sa (M.M.S.); 4 Department of Biotechnology, College of Science, Taif University, P.O. Box 11099, Taif 21944, Saudi Arabia; saqer@tu.edu.sa; 5 Department of Home Economics, College of Home Economics, King Khalid University, P.O. Box 960, Abha 61421, Saudi Arabia; hgmry@kku.edu.sa; 6 Pharmacology Department, Faculty of Medicine, Benha University, Benha 13511, Egypt; salwa.elabidine@fmed.bu.edu.eg; 7 Department of Physiology, Faculty of Veterinary Medicine, Kafrelsheikh University, Kafrelsheikh 33516, Egypt

**Keywords:** neurotoxicity, silver nanoparticles, quercetin, gene expression, immunohistochemistry, brain

## Abstract

This study aimed to investigate the oxidative neurotoxicity induced by silver nanoparticles (AgNPs) and assess the neuroprotective effects of quercetin against this toxicity. Forty adult male rats were divided into four equal groups: control, AgNPs (50 mg/kg intraperitoneally), quercetin (50 mg/kg orally), and quercetin + AgNPs. After 30 days, blood and brain tissue samples were collected for further studies. AgNP exposure increased lipid peroxidation and decreased glutathione peroxidase, catalase, and superoxide dismutase activities in brain tissue. AgNPs decreased serum acetylcholine esterase activity and γ-aminobutyric acid concentrations. AgNPs upregulated tumor necrosis factor-α, interleukin-1β, and *Bax* transcript levels. AgNPs reduced the transcripts of claudin-5, brain-derived neurotrophic factor, paraoxonase, nuclear factor-erythroid factor 2 (Nrf2), and *Bcl-2*. Histopathologically, AgNPs caused various degenerative changes and neuronal necrosis associated with glial cell reactions. AgNPs increased the immunohistochemical staining of glial fibrillary acidic protein (GFAP) in the cerebrum and cerebellum. Oral treatment with quercetin efficiently counteracted the opposing effects of AgNPs on brain tissue via modulation of tight junction proteins, Nrf2, and paraoxonase, and its positive mechanism in modulating pro-inflammatory cytokines and the downregulation of GFAP expression, and the apoptotic pathway. AgNPs also altered the severity of histopathological lesions and modulated GFAP immunostaining in the examined tissue.

## 1. Introduction

Nanotechnology has developed many nanoparticles that are very significant in medicine, industry, and agriculture. Silver nanoparticles (AgNPs) are broadly utilized in different industrial applications because of their small size and unique physicochemical features [1]. AgNPs have been employed in clothing, water disinfectants, and cosmetics because of their antibacterial and antifungal properties, and appear in air freshener sprays, wound dressings, sunscreens, hygiene products, and food containers because of their appropriate antibacterial/antifungal characteristics [2].

AgNPs can enter the body by various routes, such as the inhalation of industrial manufacturing dust or shoe sprays, injection, ingestion, and dermal contact. As a result, silver concentrations in multiple organs increase dose-dependently [3]. AgNPs can enter the bloodstream and spread throughout the body’s major organs, particularly the kidneys, liver, spleen, lungs, and brain [4]. It can also cross the placental barrier and the blood–brain barrier (BBB) [5]. Silver has a longer biological half-life in the central nervous system (CNS) [6]. AgNP exposure can have inflammatory, oxidative, genotoxic, and cytotoxic effects [7]. Sharma et al. [8] reported that AgNPs cause BBB dysfunction, neuronal deterioration, and astrocyte inflammation in rats. Moreover, severe mitochondrial shrinkage, nuclear atypia, and endoplasmic reticulum (ER) expansion have been shown in astrocytes [9]. The hippocampus’s generation of reactive oxygen species (ROS) has been linked to AgNPs [10]. ROS induce apoptosis and dark neurons. Apoptosis is a form of cell death characterized by cell shrinkage, cytoplasmic condensation, chromatin segregation, and the appearance of dark neurons (a type of cell degeneration) recognized using hyperbasophilia hyper-electron density properties [11].

Rahman et al. [12] suggested that AgNPs generate apoptosis, and neurotoxicity is caused by free radical-induced oxidative stress and gene expression changes. Authors [1] have shown that brain dopamine concentrations increased after 28 days of oral AgNP treatment (9 mg silver/kg body weight (BW)/day). Tang et al. [13] reported that AgNPs are concentrated in various tissue and organs, such as the brain, after subcutaneous injection of AgNPs into rats, leading to BBB obliteration, astrocyte bulging, and neuronal damage.

Quercetin is a natural flavonoid with numerous biological effects, including an antioxidative effect by inducing ROS-scavenging and binding to specific proteins, such as transcriptional factors and oxidative enzymes in signal-transduction pathways [14], and anti-inflammatory and neuroprotective activities [15]. Quercetin is also used in treating memory impairment [16], Huntington’s disease [17] and seizures [18]. Few facts exist about the correlation of quercetin and AgNPs in experimental models with neurotoxicity.

Because AgNPs are known to cause neurotoxicity, this study aimed to determine how quercetin protects against this toxicity. The brain is a highly sensitive organ; unsaturated fatty acids are readily peroxidizable in quercetin. Additionally, the brain does not have a particularly extensive antioxidant defense, and nerve cells are more vulnerable to poisons because of their restricted ability to renew.

## 2. Materials and Methods

### 2.1. Reagents and Chemicals

AgNP powder was provided by Sigma-Aldrich (St. Louis, MO, USA). High-intensity vortexing and sonication were performed to mix the particles with deionized water before use, to prevent particle aggregation. Solid quercetin (≥95%; high-performance liquid chromatography grade) was obtained from Sigma-Aldrich. Quercetin was dissolved in Tween-80 (0.8%, *v*/*v*). Rabbit polyclonal anti-glial fibrillary acidic protein (GFAP) antibody (ab7260) and horseradish peroxidase (HRP)/3,3′-diaminobenzidine (DAB) mouse and rabbit specific Detection IHC kit (ab64264) were purchased from Abcam (Cambridge Science Park, Cambridge, UK).

### 2.2. Experimental Design

**Figure 1:**
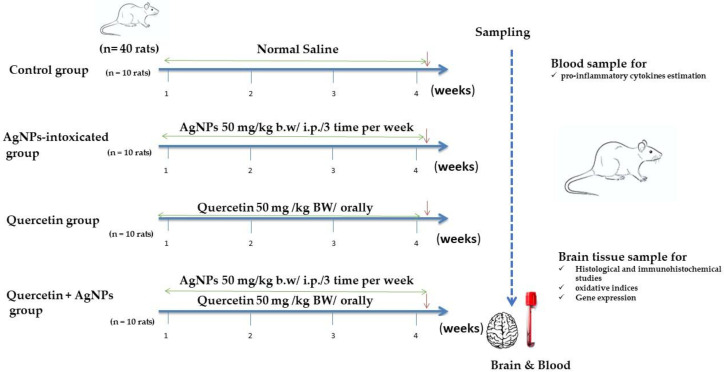
Experimental procedure.

Wistar Albino rats (160–190 g) were bought from the Animal Breeding Unit, Medical Research Institute, Alexandria University. Rats were housed in metal cages with optimal temperature and humidity, a dark/light cycle, and free feed and drinking water access. The worldwide ethical norms for the care and use of laboratory animals were followed to manage the animals. The Experimental Animal Use and Ethics Committee of Alexandria University’s Faculty of Veterinary Medicine authorized the research techniques. Four groups of rats were randomly assigned (10 rats each): control; AgNP group (AgNPs 50 mg/kg BW intraperitoneally (i.p.) administered thrice weekly; warranted by a previous study) [19]; quercetin group (50 mg/kg BW orally) [20]; and quercetin + AgNP group (infused with AgNPs and given quercetin orally) as shown in Figure 1. All treatments were given for 30 days. Rats were anesthetized with ketamine/xylazine (7.5–10 mg/kg, 1 mg/kg i.p.) 24 h after the last treatment. The inner canthus was used to collect blood. Separation of the sera for the estimation of interleukin (IL)-6 and tumor necrosis factor-α (TNF-α) pro-inflammatory cytokine levels were measured using specific rat IL-6 DuoSet ELISA Kit (DY506; R&D System, Minneapolis, MN, USA) and TNF-α Quantikine ELISA Kit (RTA00; R&D Systems, Minneapolis, MN, USA), according to the manufacturer’s instructions.

Rats were subsequently euthanized, and their brains were dissected directly, drenched in normal cold saline (0.9%), and divided into three equal portions. The first section was used for histological and immunohistochemical studies, whereas the second section assessed oxidative indices. The third section was used for gene expression analyses. Quimica Clinica Aplicada S.A. provided the acetylcholine esterase (AChE) assay kit, and γ-aminobutyric acid (GABA) was provided by Abnova.

### 2.3. Antioxidant and Lipid Peroxidation Analysis

A Teflon and a pestle homogenizer were used to homogenize 500 mg of the entire brain tissue in an ice-cold solution (0.1 M phosphate-buffered saline (PBS), pH 7.4). The supernatant was separated after centrifuging the crude homogenate at 14,000 rpm for 10 min at 4 °C; the supernatant was separated. After the reaction with thiobarbituric acid, lipid peroxide was measured and expressed as nmol malondialdehyde (MDA) per tissue weight [21]. The activity of glutathione (GSH) peroxidase (GPx) was assessed [22], based on the hydrogen peroxide (H_2_O_2_) interaction with NADPH, GSH, and GSH reductase. At a wavelength of 340 nm, the absorbance was measured, and the result was reported as IU per tissue weight. Superoxide dismutase (SOD) activity was determined based on a 50% suppression of nitroblue tetrazolium reduction by superoxide anion generated by xanthine and xanthine oxidase; the result was reported as IU per tissue weight [23]. The oxidation rate was used to determine catalase (CAT) activity according to Chiang et al. [24] and reported as IU per tissue weight. The GSH level was assessed according to Ellman [25]. GSH interacted with 5.5-dithiobis 2-nitrobenzoic acid to produce a yellow-green color and was colorimetrically quantified at 410 nm and reported as nmol per tissue weight.

### 2.4. Real-Time Polymerase Chain Reaction (RT-PCR)

The relative expression of brain tissue genes was determined using quantitative RT-PCR. Total RNA of ~100 mg brain tissue was extracted using Trizol reagent (Invitrogen, Life Sciences, Carlsbad, CA, USA) and NanoDrop for quantification. Samples of ≥1.8 A260/A280 RNA were used for DNA synthesis using cDNA synthesis (Fermentas, Waltham, MA, USA). Using the *β-actin* and *glyceraldehyde 3-phosphate dehydrogenase* household genes, the SYBR Green Master Mix and primers in Table 1 were used to enhance the cDNA. Amplification data were examined using the 2^−ΔΔT^ method [26].

### 2.5. Histopathological Examination and Scoring System

Brain tissue specimens (cerebrum and cerebellum) were taken from separate rat groups (n = 5) following necropsy, washed in saline solution, and then put in 10% buffered formalin for at least 24 h (pH 7.4). Fixed tissue specimens were embedded in paraffin, sectioned at 4.5 µm, and stained with Mayer’s hematoxylin and eosin (HE). A light microscope was used to examine the stained sections, and a digital camera was used to picture them (Nikon Corporation Co., Ltd., Tokyo, Japan). Five slides of five different rats per group were analyzed to evaluate and grade the various pathological changes found in the brain tissues (cerebrum and cerebellum). A four-point grading system was used to represent the severity of the identified histopathological changes, as follows: (—) Normal regular histology; (x) a mild injury; (xx) Moderate injury; (xxx) Severe injury [10]. 

The number of the affected slides and the number of regions impacted within each slide determined the extent of the pathological lesions in various groups. Each animal’s score was evaluated, and the mean score was calculated for the various pathological alterations per group [10].

### 2.6. Immunohistochemical Studies

For glial fibrillary acidic protein (GFAP) detection as an indicator for astrogliosis, 5-mm-thick paraffin tissue slices of the cerebrum and cerebellum were placed on positively charged slides, deparaffinized in xylene, and rehydrated in ethanol in descending concentrations. Citrate buffer (10 mM; pH 6.0) was boiled at 105 °C for 10–20 min for microwave-assisted antigen retrieval and cooled for 20 min at room temperature. Endogenous peroxidase was deactivated by 3% H_2_O_2_ in absolute methanol for 5 min at room temperature after washing with PBS. The nonspecific reaction was inhibited by incubation for 60 min with 10% normal goat serum. Rabbit polyclonal anti-GFAP antibody (1:500 dilution) was incubated overnight at 4 °C on tissue slices. For 60 min, the sections were incubated with the Mouse and Rabbit Specific HRP/DAB (ABC) Detection IHC kit. GFAP expression was tagged with horseradish peroxidase HRP and colored with diaminobenzidine (DAB) substrate for 6–10 min to identify the antigen–antibody complex. Finally, a counterstain of Mayer’s hematoxylin was applied, followed by dehydration in absolute alcohol, cleaning, and mounting. Using a light microscope, immunoreactivity was observed as dark brown GFAP staining.

### 2.7. Morphometric Investigation

Images of 10 non-overlapped high-power microscopic fields (×400) per section (one section/rat, 5 rats/group) taken with a digital camera (EC3, Leica, Munich, Germany) coupled to a Leica microscope were used to quantify GFAP expression in the cerebrum and cerebellum of control and treated groups (DM500). 

The area percent of GFAP positive brown immune-stained cells was calculated from the digital pictures using Image J analysis software (Image J 1.47v, National Institute of Health, Bethesda, MD, USA). 

### 2.8. Statistical Analysis

Using SPSS version 22.0 for Windows as a statistical package (IBM, Armonk, NY, USA), data were analyzed using a one-way analysis of variance, followed by Tukey’s *post hoc* multiple comparisons test. The obtained values are assessed as the mean ± standard error. The degree of significance was set at *p* < 0.05.

## 3. Results

### 3.1. Serum Pro-inflammatory Cytokines

**Figure 2:**
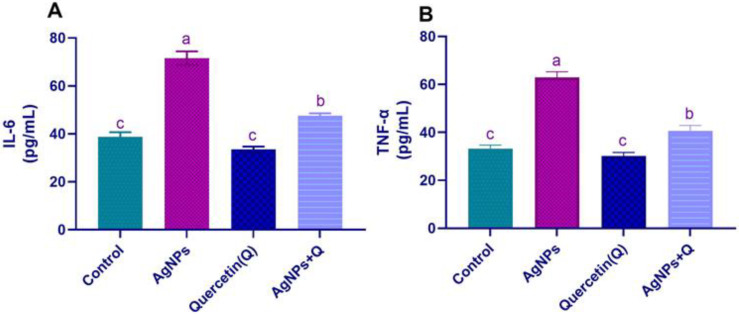
Effect of quercetin and/or silver nanoparticles on concentrations of the pro-inflammatory cytokines in rats (**A**). IL-6. (**B**). TNF-α Significantly differing mean values are indicated by different letters in the same column (*p* < 0.05). IL6, Interleukin-6; TNF-α, tumor necrosis factor-alfa; AgNPs, silver nanoparticles.

The pro-inflammatory cytokine concentrations were assessed to examine the potential neurotoxic effects of AgNPs. AgNPs induced a significant (*p* < 0.05) increment in TNF-α and IL-6 concentration compared with the control. Cotreatment of quercetin significantly (*p* < 0.05) decreased the TNF-α and IL-6 concentrations compared with that of the AgNP group, verifying that quercetin has anti-inflammatory activity, as shown in Figure 2.

### 3.2. Brain Oxidative/Antioxidative Indices

**Figure 3:**
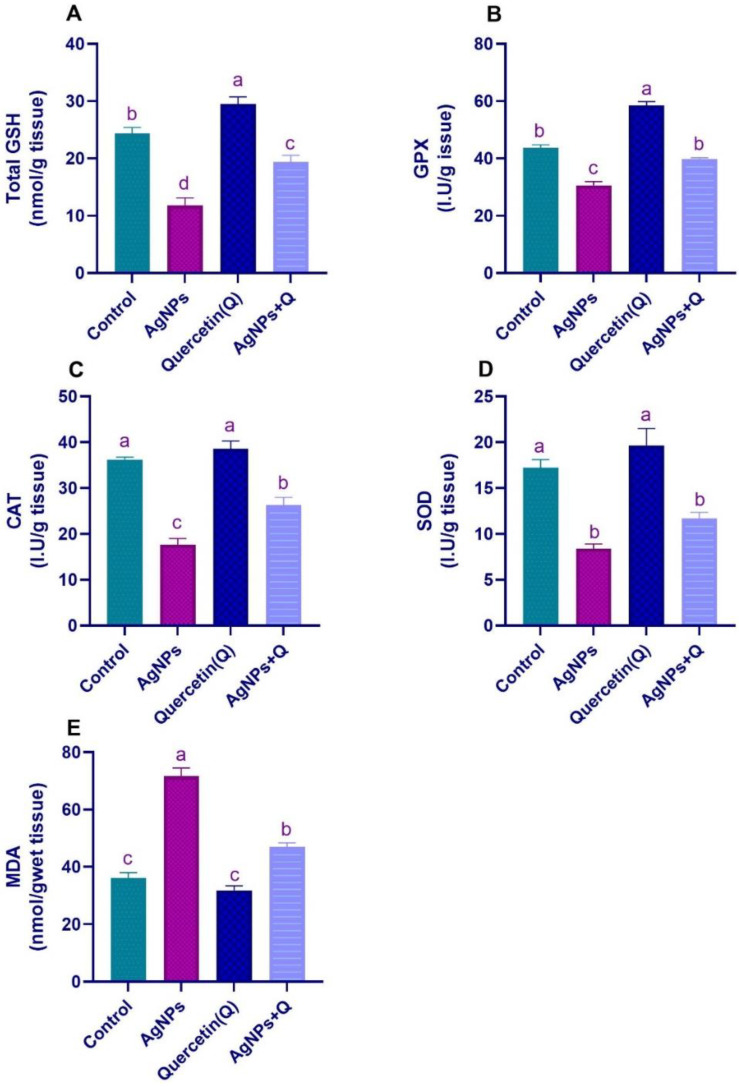
Effect of quercetin on the antioxidant markers in the brain of rats exposed to silver nanoparticles. (**A**) Total GSH, (**B**) GPX, (**C**) CAT, (**D**) SOD, (**E**) MDA. Significantly differing mean values are indicated by different letters in the same column (*p* < 0.05).

Figure 3 shows that, compared with control, AgNPs induced a significant (*p* < 0.05) increase in the MDA quantity, and a reduction in GSH levels and antioxidant enzyme activity (GPx, CAT, and SOD) in the AgNP-treated group, confirming brain tissue injury due to oxidative damage and reduction of antioxidants in this group. Compared with AgNP-treated rats, cotreatment with quercetin improved the brain oxidative/antioxidative indices, as demonstrated by a significant (*p* < 0.05) reduction in MDA and increases in different antioxidant enzymes, indicating the antioxidant effects of quercetin.

### 3.3. Brain Neurotransmission

**Figure 4:**
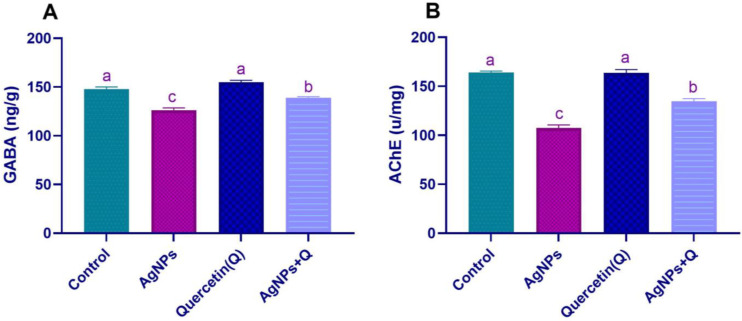
Effect of quercetin on GABA (**A**) concentration and AChE (**B**) enzymatic activity in silver nanoparticle-exposed rats’ brain homogenates. Significantly differing mean values are indicated by different letters in the same column (*p* < 0.05)—AgNPs, silver nanoparticles; GABA, gamma-Aminobutyric acid; AchE, acetylcholine esterase.

GABA amino acid neurotransmitter mediates fast excitatory and inhibitory neurotransmission in the brain, and ACE is a very fast enzyme that terminates neurotransmission at cholinergic synapses. The brain enzymatic activity of AChE and GABA concentrations is shown in Figure 3. Compared with control, AgNPs treatment induced a significant decrease in (*p* < 0.05) AChE activity, and GABA concentrations confirmed the disturbance in neuroendocrine control that led to the destructive neuronal consequences of AgNPs. Quercetin coadministration reduced the biochemical assays compared with the AgNP group, as demonstrated by a significant (*p* < 0.05) increase, as shown in Figure 4.

### 3.4. Lesion Grading and Histopathological Findings

The average scores of pathological lesions in the cerebrum and cerebellum of different rat groups are shown in Table 2. The control and quercetin-treated groups showed regular histological limits. AgNP-treated rat tissue exposed histological changes that are quite severe, and had a high score in all examined characteristics. Nevertheless, cotreatment of quercetin with AgNPs improved most of these changes and revealed mild to moderate pathological abnormalities.

**Figure 5:**
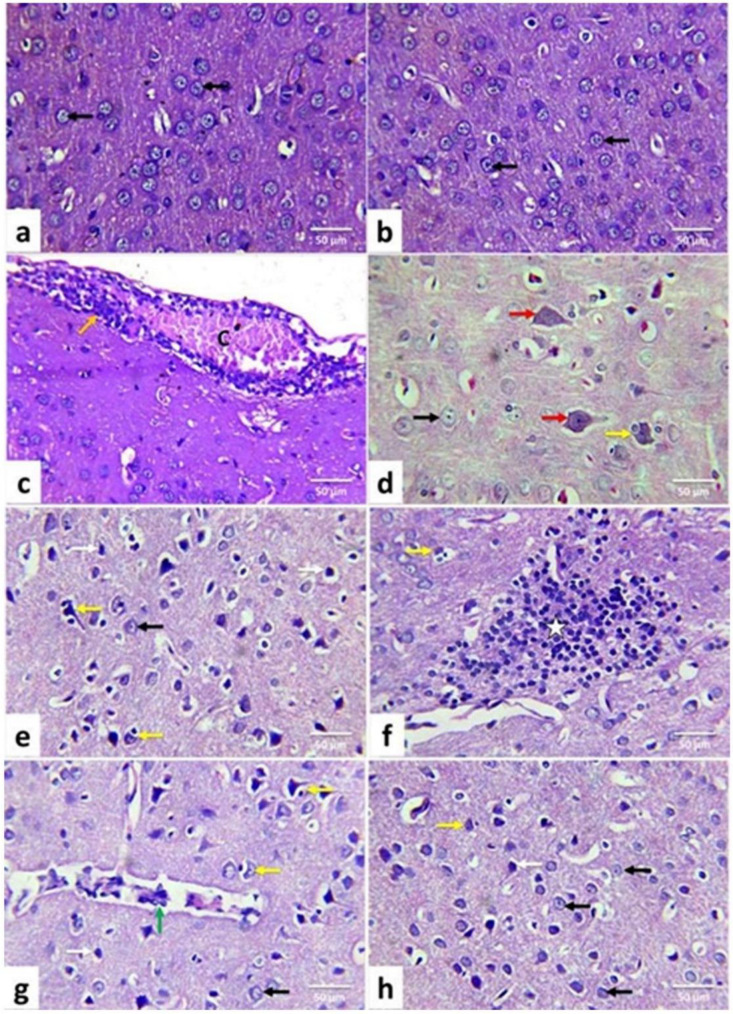
Illustrative photomicrograph of rat cerebral cortex (HE, ×400). Control (**a**); Quercetin-treated (**b**); Silver nanoparticles-treated (**c**–**h**) silver nanoparticles + quercetin-treated groups. Normal neurons (black arrow); vascular congestion (C) mononuclear inflammatory cells infiltrations (orange arrow); swollen neurons (red arrow); shrunken darkly stained neurons (white arrow); the area with gliosis (star); necrotic neurons with satellitosis and neuronophagia (yellow arrow); perivascular cuffing (green arrow).

**Figure 6:**
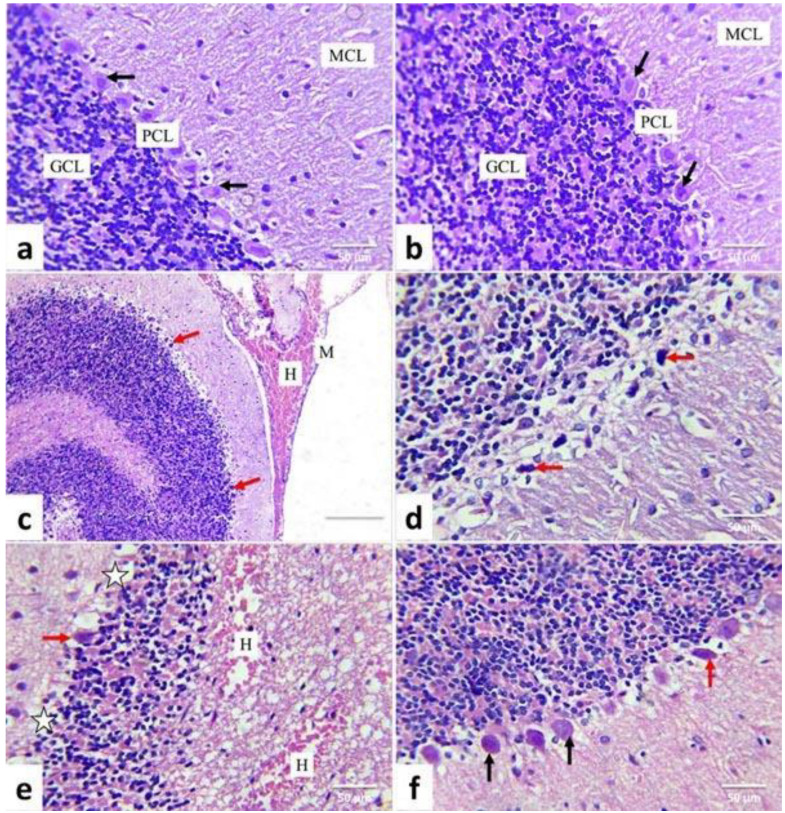
Illustrative photomicrograph of rat cerebellum (HE, ×400). Control (**a**); Quercetin- treated (**b**); Silver nanoparticles-treated (**c**–**f**) silver nanoparticles + quercetin-treated groups. Meninges (M); molecular cell layer (MCL); Purkinje cell layer (PCL) and granule cell layer (GCL); normal Purkinje cells (black arrow); Shrunken darkly stained Purkinje cell with pyknotic nuclei (red arrow); hemorrhage (H) loss of Purkinje cell (star).

The control and quercetin-treated rats revealed nearly normal histological structures of the cerebrum cortices (Figure 5a,b) and cerebellum (Figure 6a,b). In AgNP-treated rats, cerebral tissue showed meningitis, accompanied by congestion of blood vessels, edema, hemorrhage, and mononuclear inflammatory cell infiltrations (Figure 5c). Many cortical neurons displayed neuronal swelling (Figure 5d); others were shrunken and intensely stained, associated with increased pericellular spaces. Necrotic neurons had pyknotic nuclei and hypereosinophilic cytoplasm with or without satellitosis and neuronophagia (Figure 5e). The neuropil also displayed variable-size vacuoles and focal areas of gliosis (Figure 5f). 

Additionally, perivascular cuffing (Figure 5g), congestion, and areas of hemorrhages have been observed.

Meninges displayed vascular congestion, edema, hemorrhage (Figure 6c), and infiltration of mononuclear inflammatory cells in the cerebellum. The Purkinje layer was separated from the rest of the brain’s layers. Many Purkinje cells with pyknotic and hyperchromatic nuclei were observed (Figure 6d). Others were necrotic and associated with satellitosis and neuronophagia. A selective neuronal loss was also observed. Cellular depletion was evident in the granular cell layer. Also, areas of gliosis, congestion, and hemorrhages (Figure 6e) were observed. In comparison, AgNPs + quercetin-treated rats’ cerebrum cortices (Figure 5f) and cerebellum tissues (Figure 6f) displayed a significant improvement in the previously observed lesions in AgNPs-treated rats as they were less in severity and distribution (Table 2). 

### 3.5. Immunohistochemical Analysis and Quantitative Assessment

**Figure 7:**
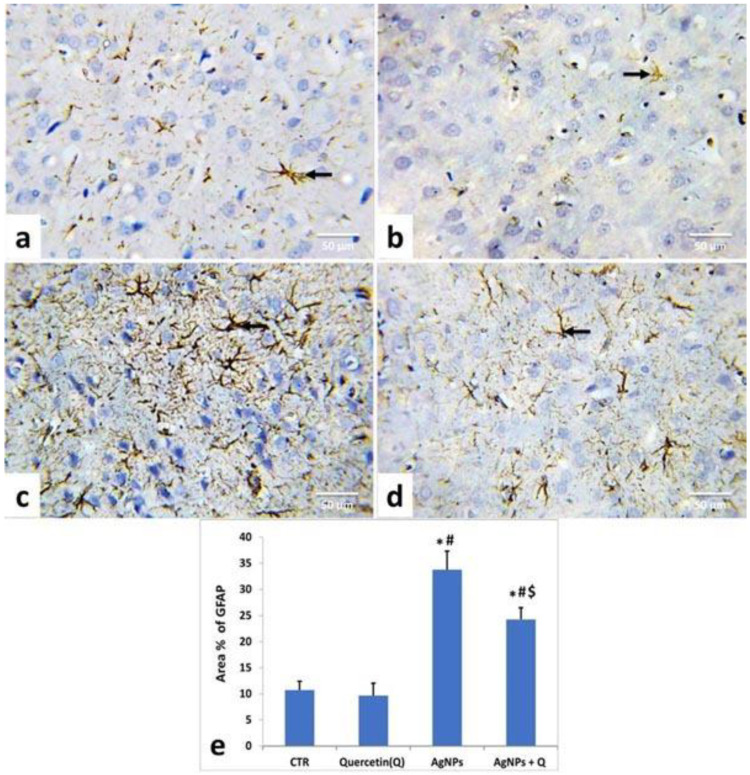
Illustrative photomicrograph showing the distribution and activation of astrocytes in the cerebral cortices of different groups using immunohistochemical staining for GFAP (IHC, ×400). Control (**a**); Quercetin-treated (**b**); Silver nanoparticles-treated (**c**) silver nanoparticles + quercetin-treated groups (**d**). Arrow represents the astrocytes; (**e**) Quantification of GFAP expression, the immunohistochemical staining of GFAP was assessed as area (%) across 10 fields/section, n = 5 rat/group. Mean values were significantly different from control (# *p* < 0.05), Quercetin (* *p* < 0.05), silver nanoparticles ($ *p* < 0.05) group.

**Figure 8:**
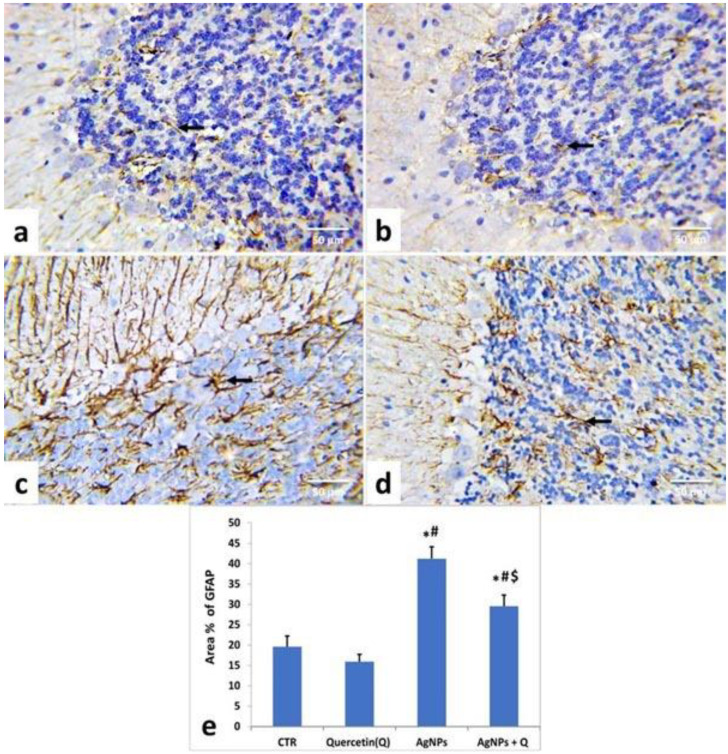
Illustrative photomicrograph demonstrating astrocytes’ distribution and activation in the cerebellar tissues of different groups using immunohistochemical staining for GFAP (IHC, ×400). Control (**a**); Quercetin-treated (**b**); Silver nanoparticles-treated (**c**) silver nanoparticles + quercetin-treated groups (**d**). Arrow represents the Astrocytes; (**e**) Quantification of GFAP expression, the immunohistochemical staining of GFAP was assessed as area (%) across 10 fields/section, n = 5 rat/group. Mean values were significantly different from control (# *p* < 0.05), Quercetin (* *p* < 0.05), silver nanoparticles ($ *p* < 0.05) group.

As demonstrated in Figure 7 and Figure 8, the immunohistochemical staining of the cerebral cortices and the cerebellum of the control (Figure 7a and Figure 8a) and quercetin-treated (Figure 7b and Figure 8b) groups showed brown-yellow small astrocyte cell bodies with thin processes. AgNPs-treated rats’ tissues displayed relatively larger cells with prominent processes (Figure 7c and Figure 8c). 

In contrast, AgNP + quercetin-treated rats significantly reduced astrocytic reactions (Figure 7d and Figure 8d). In line with these remarks, quantitative assessment of the GFAP-immunostained area percentage in the cerebrum and cerebellum of quercetin-treated rats presented no significant (*p* < 0.05) changes compared with those of control rats (Figure 7e and Figure 8e, respectively). Meanwhile, AgNPs-treated rats’ tissues displayed a substantial (*p* < 0.05) increment as contrasted to the control group values (Figure 7e and Figure 8e). Conversely, AgNP + quercetin-treated rats displayed a significant (*p* < 0.05) decrease in the GFAP area percentage.

### 3.6. Effects of Quercetin and/or AgNP on Gene Expression

**Figure 9:**
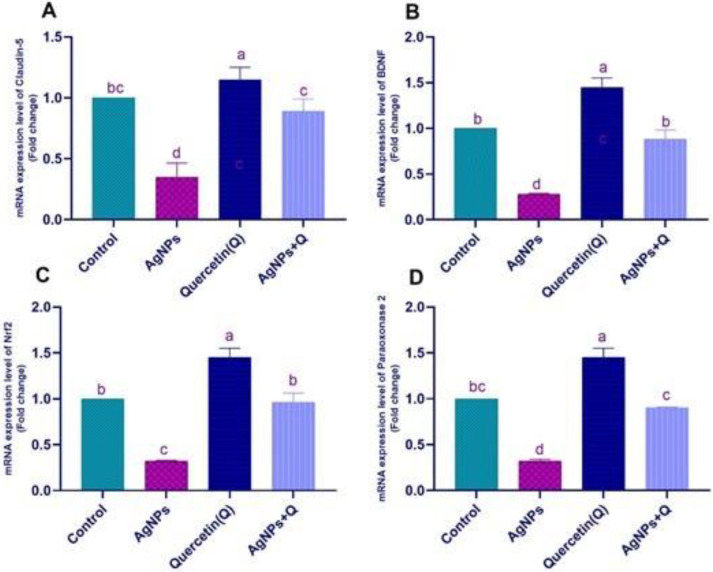
The protective effect of quercetin against AgNPs induced changes in the expression of Claudin-5 (**A**), *BDNF* (**B**), Nrf2 (**C**), and Paraoxonase-2D (**D**). Values are means ± SEM for seven different rats per treatment. Statistically significant values have different letters at *p* < 0.05.

**Figure 10:**
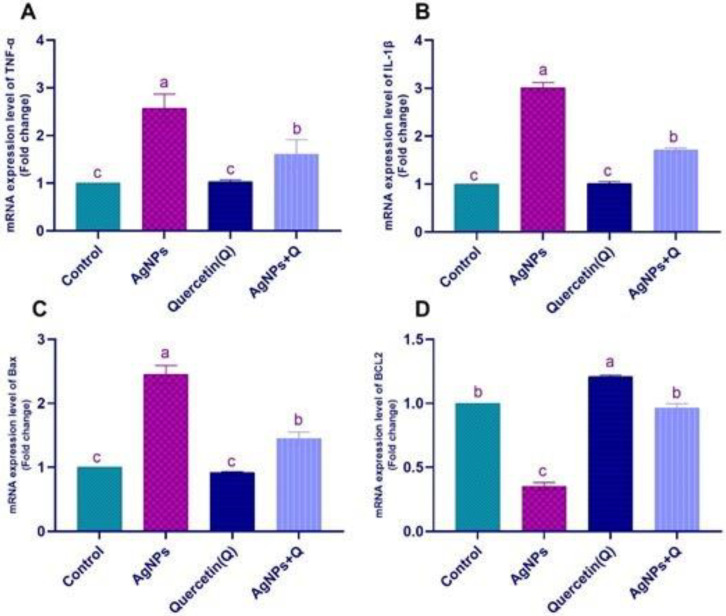
The protective impact of quercetin against AgNPs persuaded alteration of the rat renal TNF-α (**A**), *IL-1β* (**B**), *Bax* (**C**), and Bcl2 (**D**) expression. Values are means ± SEM for 10 different rats per treatment. Statistically significant values have different letters at *p* < 0.05.

In Figure 9, AgNPs significantly downregulated the claudin-5 and brain-derived neurotrophic factor (*BDNF*) mRNA gene expression and the nuclear factor-erythroid factor 2 (NrF2) and paraoxonase mRNA gene expression. Quercetin cotreated with AgNPs showed a significant upregulation of claudin-5. The AgNP group showed significant normalization of NrF2 and paraoxonase mRNA expression. In Figure 10, AgNPs significantly upregulated the TNF-α, *IL-1β*, and *Bax* mRNA expression, with significant downregulation of the *Bcl-2* expression that significantly normalized with quercetin treatment.

## 4. Discussion

The brain is a susceptible organ; it is vulnerable to the effects of ROS, owing to the high demand for oxygen, the highly peroxidizable unsaturated fatty acids, and the abundance of highly peroxidizable substrates [27]. Moreover, the brain’s antioxidant defenses are not particularly generous because of their restricted ability to repair nerve cells that are more vulnerable to toxins [28]. AgNPs have been regularly utilized for many years in consumer products. Cell death can be caused by various methods, such as exposure to high ROS quantities generated by AgNPs [29]. This study aimed to investigate the neuroprotective effects of quercetin on male adult rats exposed to AgNPs.

Oxidative stress leads to BBB disturbance parallel to the changes in cytoskeletal tight junction proteins (TJPs), such as claudin-5 and occludin [30]. Smaller AgNPs can easily penetrate the BBB, a detriment to the barrier’s integrity by modifying the cell membrane’s endothelial permeability [9]. The deposition of silver over a long time leads to significant brain dysfunction [31]. The BBB plays an essential role in the pathogenic progression [32]. Results revealed that AgNPs significantly downregulated claudin-5 and *BDNF* mRNA gene expression, inconsistent with Martirosyan et al. [33], who reported that occludin in AgNP-treated cells was disturbed. This study was consistent with Park et al. [34], who reported that AgNPs substantially inhibited *BDNF*-induced cell survival by increased lactate dehydrogenase secretion and oxidative ROS creation. This study showed that quercetin cotreated with AgNPs normalized claudin-5 and *BDNF* mRNA expression. This result was consistent with [30,35], who revealed that quercetin preserves and avoids hippocampus neuronal injury through upregulation of the TJPs of the BBB and *BDNF*. Quercetin significantly normalized NrF2 and paraoxonase mRNA expression. Nrf2 attaches to the antioxidant/electrophilic response factor and governs cytoprotective protein expression, mainly antioxidant GSH producing enzymes [36]. MDA levels were lower, and antioxidant enzyme activity was higher in AgNP-intoxicated rats given quercetin. This study was supported by Xiao et al. [37], who revealed that quercetin decreases brain cell apoptosis and neuronal injury by improving pathways of DJ-1/Nrf2 and enhancing the possibility of antioxidation. In addition, Pereira et al. [38] showed that paraoxonase is related to several oxidative stress diseases. Paraoxonase 2 has anti-inflammatory and neuroprotective properties [39,40]. Costa et al. [41] showed the neuroprotection effects of quercetin through modulation of paraoxonase 2 expressions. AgNPs significantly upregulated TNF-α, *IL-1β*, and *Bax* mRNA expression, with significant downregulation of *Bcl-2* expression that significantly normalized with quercetin treatment.

This study was consistent with Murphy et al. [42], who proved that, in primary blood monocytes, AgNPs could massively increase the mRNA expression of the pro-inflammatory cytokines IL-1, IL-6, and TNF-α, which provoked the inflammatory and oxidative effect of AgNPs. Others [43] also showed that quercetin and MSG normalized inducible nitric oxide synthase, *IL-1β*, and TNF-α. AgNPs decreased *Bcl-2* and increased *Bax* gene expression in hippocampal rat cells [43], supporting the findings on apoptosis. Additionally, [43] found that quercetin has a defensive role by partly controlling *Bcl-2*/*Bax* protein expression. Moreover, AChE plays a crucial role in neuromuscular impulse transmission and is considered a potential biomarker of xenobiotic toxicity [10]. In this study, AChE activity was inhibited by AgNPs, consistent with Myrzakhanova et al. [44], who reported that toxicity with AgNPs leads to acetylcholine decrease.

Increased expression of acetylcholine leads to increased ROS and inflammation. GABA is an inhibitory neurotransmitter. When GABA attaches to a GABA receptor, it produces a calming effect [45]. This study reported that AgNPs induced a significant reduction in GABA concentrations, confirming the disturbance in neuroendocrine control that led to the neuronal damaging effects of AgNPs.

This study was supported by Jakaria et al. [46], who revealed that quercetin has a significant anxiolytic activity mediated by the GABAergic nervous system. Therefore, from the aforementioned results, it can be concluded that the protective pathway of quercetin against AgNP toxicity is through the modulation of TJPs, Nrf2, and paraoxonase, and its positive mechanism in modulating the pro-inflammatory cytokines and the apoptotic pathway.

Histological and immunohistochemical studies confirmed the biochemical findings. Various histological abnormalities (i.e., glial cell activation) in the AgNP-treated rat cerebrum and cerebellum were related to neuronal degeneration and necrosis, consistent with previous findings [47,48]. The capacity of AgNPs to cause oxidative stress, ROS production, mitochondrial dysfunction, and cytoskeleton alterations may be due to these lesions and impaired DNA and protein synthesis, resulting in inflammation, cell necrosis, or apoptosis [49,50].

ROS interacts with proteins, DNA, and RNA, among other biological elements. Vital cellular functions are disrupted. Furthermore, free radicals target the cell membrane polyunsaturated fatty acids and trigger the cell membrane’s lack of permeability and disintegration [10]. ROS can potentially affect brain vascular functions, resulting in cell death [51]. AgNPs also decrease the interference with the synaptic machinery [9,52,53] and disruption of the BBB [52,53].

GFAP is a type III intermediate filament protein that maintains the astrocyte shape and mechanical strength [54]. Modifying the cell’s filament network plays a role in several key activities in the CNS, including cell communication, support for nearby neurons, BBB functions, and mitosis [55]. GFAP serves as a specific marker of astrocytic activity and, during astrogliosis, is significantly expressed in CNS astrocytes [47]. Reactive astrogliosis may impair neuronal survival [56]. Glial cells create neurotoxic chemicals that cause inflammation and neuronal death, for example, reactive oxygen radicals and excitatory amino acids [57].

GFAP immunohistochemical staining in the cerebrum and cerebellum showed that AgNP-treated rats had a high level of GFAP positivity. The cerebrum and cerebellum showed an upsurge in the area percentage of GFAP-positive cells in the quantitative analysis of the AgNP-treated group, consistent with Yin et al. [58]. Reactive astrogliosis may impair neuronal survival [56] by the release of many neurotoxic chemicals (including reactive oxygen radicals and excitatory amino acids) and the consequent induction of inflammation and neuronal death by glial cells [57]. AgNPs may promote reactive gliosis and inflammation by excessive induction of lipid peroxidation, inducing oxidative stress and depletion of antioxidants that enhance the incidence of inflammation [59].

Concurrent administration of quercetin with AgNPs partially ameliorated these histopathological changes, as demonstrated by histopathological scoring. Correspondingly, quercetin downregulated GFAP expression in brain tissue. These data were in line with those of previous studies that reported the ability of quercetin to mitigate histological changes in the brain tissue of rats after traumatic brain injury [60] and various neurotoxicants [61,62,63]. The neuroprotective effects of quercetin might be ascribed to its aptitude to ameliorate oxidative damage, avoid lipid peroxidation, mitigate ER stress, modulate microglial activation [64,65,66], protect against AgNP-induced free radical assault, and boost the overall antioxidant capacity.

## 5. Conclusions

Quercetin administration effectively counteracted the adverse effects of AgNPs via modulation of TJPs, Nrf2, and paraoxonase and the expression of pro-inflammatory cytokines and the GFAP apoptotic pathway.

## Figures and Tables

**Table 1:** Primers for RT-PCR.

Gene	Direction	Sequence	Accession Number
*Bax*	Sense	GGCGAATTGGCGATGAACTG	NM_017059.2
	Antisense	ATGGTTCTGATCAGCTCGGG	
*Bcl-2*	Sense	GATTGTGGCCTTCTTTGAGT	NM_016993.1
	Antisense	ATAGTTCCACAAAGGCATCC	
*Cldn5*	Sense	CGTGACGGCGCAGACGACTT	NM_031701.2
	Antisense	TGCACTGAGCGCCGGTCAAG	
*BDNF*	Sense	TTGCCACAGCCCCAGGTGTGA	NM_012513.3
	Antisense	ACGCCTGTCACTGCGCCCTA	
*Tnf-α*	Sense	GTAGCCCACGTCGTAGCAAAC	NM_012675.3
	Antisense	ACCACCAGTTGGTTGTCTTTGA	
*Nrf2*	Sense	CCCATTGAGGGCTGTGATCT	NM_031789.2
	Antisense	GCCTTCAGTGTGCTTCTGGTT	
*IL-1β*	Sense	TGACAGACCCCAAAAGATTAAGG	NM_031512.2
	Antisense	CTCATCTGGACAGCCCAAGTC	
*iNOS*	Sense	GTTCCCCCAGCGGAGCGATG	NM_012611.3
	Antisense	ACTCGAGGCCACCCACCTCC	
*paraoxonase 2*		CGCCACCAATGACCACTACT	NM_001013082
		TTGATCCCGTTGGCTGAGTC	
*β actin*	Sense	TCCACCCGCGAGTACAACCTTC	NM_031144.2
	Antisense	GGGCCACACGCAGCTCATTGTA	
*GAPDH*	Sense	TCAAGAAGGTGGTGAAGCAG	NM_017008.4
	Antisense	AGGTGGAAGAATGGGAGTTG	

*Bax*, *Bcl-2*-associated X protein; *Bcl-2*, B-cell lymphoma 2; *GAPDH*, glyceraldehyde-3-phosphate dehydrogenase; *Cldn5*, Claudin-5; *BDNF*, Brain-derived neurotrophic factor; *Tnf-α*, Tumor necrosis factor; *Nrf2*, Nuclear factor-erythroid factor 2; *IL-1β*, interleukin I beta; *iNOS*, Inducible nitric oxide synthase.

**Table 2:** Cerebral and cerebellar lesion grading of control (CTR), quercetin and/or silver nanoparticles-treated rats.

Organ	Lesions	Control	Quercetin	AgNPs	AgNPs + Quercetin
Cerebral cortex					
	Blood vessels congestion				
	Meninges	—	—	xx	x
	Cerebral cortex	—	—	x	x
	Neuronal pyknosis	—	—	xxx	xx
	Intracellular and extracellular vacuolization	—	—	xx	x
	Glial cell reaction	—	—	xxx	xx
	Cerebral hemorrhage	—	—	x	—
	Perivascular cuffing	—	—	xx	x
Cerebellar cortex					
	Blood vessels congestion				
	Meninges	—	—	xx	x
	Granular layer	—	—	x	—
	Pyknosis of Purkinje cell	—	—	xxx	xx
	Perineural vacuolation	—	—	xx	x
	Necrosis of Purkinje cell	—	—	xxx	xx
	Loss of Purkinje cells	—	—	xx	x
	Depletion of the granule cell layer	—	—	x	—
	Hemorrhage	—	—	xx	x

(—) Normal regular histology; (x) A minor injury; (xx) Moderate injury; (xxx) Severe injury.

## Data Availability

All data sets obtained and analyzed during the current study are available in the manuscript.
